# The β-hydroxybutyrylation of Zyxin ameliorates pulmonary fibrosis by inhibiting lung fibroblast activation through the PI3K/AKT pathway

**DOI:** 10.1186/s12931-026-03732-0

**Published:** 2026-05-30

**Authors:** Li Qiu, Haoying Huang, Sen Li, Haixia Chen, Xiaohui Wang

**Affiliations:** 1 Guangzhou Women and Children’s Medical Center, State Key Laboratory of Respiratory Disease, Guangdong Basic Research Center of Excellence for Respiratory Medicine, Guangzhou Medical University, Guangzhou, 510623 China; 2 Translational Medical Center for Stem Cell Therapy, Institute for Regenerative Medicine, Shanghai East Hospital, School of Life Sciences and Technology, Tongji University, Shanghai, 200127 China; 3 Shanghai Key Laboratory of Signaling and Disease Research, Frontier Science Center for Stem Cell Research, School of Life Sciences and Technology, Tongji University, Shanghai, 200092 China

**Keywords:** β-hydroxybutyrylation, Pulmonary fibrosis, Fibroblast activation, PI3K/AKT pathway, Zyxin, β-hydroxybutyrate supplementation

## Abstract

Idiopathic pulmonary fibrosis (IPF) is a lethal interstitial lung disease with poorly understood pathogenesis. Here, we identify reduced lysine β-hydroxybutyrylation (Kbhb) of the focal adhesion protein Zyxin in bleomycin (BLM)-induced pulmonary fibrosis in mice. In a BLM-induced mouse model, we observe a significant reduction in lung tissue Kbhb levels, while β-hydroxybutyrate (β-OHB) supplementation restores Kbhb modification, there by mitigating fibrosis. Transcriptome profiling suggests that β-OHB exerts anti-fibrotic effects by modulating the PI3K/AKT pathway. Proteomic analysis further reveals a decrease in Kbhb modification at the K263 site of Zyxin in IPF mice. In vivo experiments demonstrate that Zyxin knockout or β-OHB treatment markedly alleviates fibrotic pathology and reduces collagen deposition as well as mouse mortality. This study is the first to elucidate the mechanism by which Zyxin Kbhb modification suppresses pulmonary fibrosis via the PI3K/AKT pathway, offering a novel metabolic modification-based therapeutic strategy for IPF.

**Supplementary Information:**

The online version contains supplementary material available at 10.1186/s12931-026-03732-0.

## Introduction

Idiopathic pulmonary fibrosis (IPF) is a progressive and lethal interstitial lung disease, with a median survival of only 3–5 years post diagnosis [1]. This devastating prognosis, coupled with rising global incidence and mortality rates, underscores an urgent unmet medical need. Pulmonary fibroblasts are the primary effector cells driving IPF pathology. Aberrant activation of pulmonary fibroblasts, triggered by repetitive alveolar epithelial injury and dysregulated repair, leads to excessive extracellular matrix (ECM) deposition, chronic inflammation and progressive destruction of lung architecture, culminating in respiratory failure [2]. The core challenge in effectively treating IPF lies in our limited understanding of its pathogenic mechanisms, resulting in the lack of therapeutic options with often suboptimal efficacy [3]. Therefore, elucidating the pathogenesis of IPF and identifying new therapeutic targets are of great scientific and clinical significance for developing effective treatments and prolonging the life span of patients.

Post-translational modifications (PTMs) represent a crucial mechanism for regulating protein function, localization, stability and interactions, achieved through enzymatic covalent additions to specific amino acid residues [4, 5]. Dysregulation of PTMs is increasingly implicated in diverse pathologies, including metabolic disorders [6], cancer [7, 8], and cardiovascular diseases [9], positioning PTM research as a vital frontier for uncovering disease mechanisms and therapeutic targets. While PTMs like acetylation that is involved in fibroblast differentiation and ubiquitination that affects pro-fibrotic macrophage function have been implicated in IPF, the comprehensive landscape of pathogenic PTM alterations in IPF [10–12], particularly within activated fibroblasts, remains largely unexplored. This knowledge gap necessitates the investigation of novel PTMs and their functional roles in IPF pathogenesis to identify new therapeutic avenues. In recent years, lysine β-hydroxybutyrylation (Kbhb) has emerged as a novel and metabolically-sensitive PTM, directly bridging cellular metabolic state to epigenetic and functional regulation [13]. Kbhb is characterized by the covalent attachment of β-hydroxybutyrate (β-OHB), a canonical ketone body, to the ε-amino group of lysine residues. This reaction, catalyzed by specific acyltransferases, results in a structurally distinct modification that is significantly larger and more hydrophilic than acetylation, thereby capable of inducing profound changes in protein conformation, stability, protein-protein interactions, and subcellular localization.

Zyxin is a critical component of focal adhesion and plays vital roles in cell adhesion, migration, and proliferation [14]. Disruption or dysfunction of Zyxin may lead to impaired focal adhesion stability and aberrant mechanotransduction, contributing to diseases like cancer metastasis through loss of migratory control [15] and skin fibrotic disorders via excessive extracellular matrix deposition [16]. Despite these established connections to cellular processes and disease pathogenesis, Zyxin’s specific role in IPF development remains unknown.

In our study, we reveal a reduction in Kbhb of the focal adhesion protein Zyxin in a bleomycin (BLM)-induced pulmonary fibrosis in mice. Inhibition of Zyxin Kbhb triggers spontaneous lung fibrosis, establishing its causal role. Importantly, restoration of Zyxin Kbhb attenuates fibrosis in vivo. Our findings shed light on a potential therapeutic strategy for IPF.

## Materials/subjects and methods

### Human subjects

Lung tissue samples were obtained from patients with either bronchiolitis obliterans (BO) or congenital pulmonary cyst. Fibrotic samples were collected from BO-affected regions exhibiting pathological thickening and fibrosis, while control tissue was obtained from macroscopically normal lung parenchyma resected during the removal of congenital pulmonary cysts. BO is a chronic progressive fibrotic disease of the small airways, and all patients with BO included in this study had clinically established chronic disease with histopathological evidence of fibrosis. However, due to the retrospective nature of sample collection, precise data on individual disease duration were not available. All patient samples were provided by the Guangzhou Women and Children’s Medical Center, Guangzhou Medical University. This study was conducted in accordance with the Declaration of Helsinki and was approved by the Guangzhou Medical University College of Pharmacy Ethical Committee (Ethics number: GWCMC-2025-184A01). Written informed consent was obtained from all patients or their legal guardians.

### Mice

All animal studies were performed according to protocols approved by the Animal Ethics Committee of Guangzhou Women and Children’s Medical Center, Guangzhou Medical University. Male C57BL/6 mice (Guangdong Medical Laboratory Animal Center) aged 6–8 weeks with an average body weight of 18–23 g were used. Mice were acclimatized for one week post-arrival under standard housing conditions prior to experimentation. Mice were randomly assigned to experimental groups. To induce pulmonary fibrosis, mice received a single intratracheal administration of bleomycin (BLM) (2 mg/kg, APExBIO, Houston, USA) under anesthesia, and lung tissues were harvested for histological analysis on day 21 post-instillation [17, 18]. Control mice received an equivalent volume of phosphate buffered saline (PBS) via the same route. At a predetermined time point post-BLM/PBS administration, mice received intranasal administration of either negative control (NC) siRNA or Zyxin siRNA (Sangon Biotech, Shanghai) under isoflurane anesthesia using Entranster^TM^-in vivo transfection reagent (Engreen Biosystem Co, Ltd). siRNA sequences are provided in Supporting Information Table S1. Investigators were not blinded to group allocation during procedures and analysis. Group sizes were determined based on pilot data and previous literature to ensure sufficient statistical power to detect relevant effects.

### Cell culture and transfection

The mouse lung fibroblast cell line MLg and macrophage cell line RAW264.7 were purchased from ATCC (Manassas, VA, USA). The short tandem repeat (STR) profiling was used to test the authenticity of the cells; these cells were free of mycoplasma contamination. The cells were cultured at 37 °C in a humidified atmosphere of 5% CO_2_ in RPMI-1640 or DMEM basic medium supplemented with 10% fetal bovine serum (FBS) as a medium. For transfection, cells were seeded in 6-well plates and grown to 80% confluence before transfection using Lipofectamine 3000 (Invitrogen) according to the manufacturer’s protocol. After 24 h, cells were harvested for further analysis.

### β-hydroxybutyrate (β-OHB) administration in mice

Mice were injected with 100 µL β-OHB (100 mg/mL) intraperitoneally. Control mice were injected with PBS. The administration started at 7th and lasted for 14 d. Lung tissues were harvested for histological analysis.

### Immunohistochemistry

Lung tissues were fixed in 4% paraformaldehyde (Biosharp, Hefei, China) and embedded in paraffin to prepare 5-µm tissue sections. Sections were deparaffinated in xylene and rehydrated through a graded ethanol series to distilled water. After antigen retrieval and cooling to room temperature, endogenous peroxidase activity was quenched by incubation with 3% hydrogen peroxide (H₂O₂) in distilled water for 10–15 min. The sections were blocked with 2% Bovine serum albumin (BSA, Sigma, USA) for 1 h, followed by incubation with anti-acetyllysine rabbit mAb (Jingjie Biotechnology, Hangzhou, China), anti-β-hydroxybutyryllysine rabbit mAb (Jingjie Biotechnology), anti-succinyllysine rabbit pAb (Jingjie Biotechnology), anti-crotonyllysine rabbit pAb (Jingjie Biotechnology), anti-collagen I antibody (abcam, UK) and anti-*α*-SMA antibody (abcam) at 4 ℃ overnight. Sections were then incubated with horseradish peroxidase-conjugated secondary antibodies (Beyotime, Shanghai, China) and developed color by 3,3′-diaminobenzidine (DAB, Beyotime). Results were visualized and images captured using a bright-field microscope (DMi8, Leica, Germany).

### Hematoxylin and eosin (H&E) and Masson’s trichrome staining

Hematoxylin (Servicebio, Wuhan, China) and eosin (Beyotime) were used for Hematoxylin and Eosin (H&E) staining. Masson’s trichrome staining was performed according to the instructions of the kit (Solarbio, Beijing, China).

### Western blot

Samples were lysed in RIPA buffer containing protease inhibitor cocktail (Beyotime) and phenylmethylsulfonyl fluoride (PMSF). Equal amounts of protein (30–50 µg) were loaded and separated on a 10% SDS-polyacrylamide gel, electro-transferred onto Polyvinylidene fluoride membranes and blocked in 5% bovine serum albumin in Tris-buffered saline containing Tween 20 for 1 h at room temperature. The membranes were probed with primary antibodies overnight at 4 °C. After three 10 min washes with PBS with Tween-20 (PBST), the membrane was incubated at room temperature for 1 h with an appropriate secondary antibody conjugated to horseradish peroxidase. The protein signals were visualized with an ECL Western Blotting kit (Millipore, Massachusetts, USA). Uncropped western blots can be found in the supplement.

### Untargeted metabolomic analysis

The following procedure was performed by Magigene Technology Company (Guangzhou, China). The lung tissue samples (25 mg ± 1 mg) were taken, mixed with beads and 500 µL of extraction solution (methanol: acetonitrile: H_2_O, 2:2:1 (v/v/v)). The extraction solution contains deuterated internal standards. The mixture was vortexed for 30 s. Then the mixture was homogenized (35 Hz,4 min) and sonicated for 5 min in 4 ℃ water bath, the step was repeated for three times. The homogenate was incubated for 1 h at -40 ℃ to precipitate proteins. Then the homogenate were centrifuged at 12,000 rpm for 15 min at 4 ℃. The supernatant was transferred to a fresh glass vial for analysis. The quality control (QC) sample was prepared by mixing an equal aliquot of the supernatant of samples.

LC-MS/MS analyses were performed using an UHPLC system (Vanquish, Thermo Fisher Scientific) with a Waters ACQUITY UPLC BEH Amide (2.1 mm × 50 mm, 1.7 μm) coupled to Orbitrap Exploris 120 mass spectrometer (Orbitrap MS, Thermo Fisher Scientific). The mobile phase consisted of 25 mmol/L ammonium acetate and 25 mmol/L ammonia hydroxide in water (pH = 9.75) (A) and acetonitrile (B). The auto-sampler temperature was 4 ℃, and the injection volume was 2 µL. The Orbitrap Exploris 120 mass spectrometer was used for its ability to acquire MS/MS spectra on information-dependent acquisition (IDA) mode in the control of the acquisition software (Xcalibur, Thermo Fisher Scientific). In this mode, the acquisition software continuously evaluates the full scan MS spectrum. The ESI source conditions were set as following: sheath gas flow rate as 50 Arb, Aux gas flow rate as 15 Arb, capillary temperature 320 ℃, full MS resolution as 60,000, MS/MS resolution as 15,000, collision energy: SNCE 20/30/40, spray voltage as 3.8 kV (positive) or-3.4 kV (negative), respectively.

The raw data were converted to the mzXML format using ProteoWizard and processed with an in-house program. which was developed using R and based on XCMS, for peak detection, extraction, alignment, and integration. The R package and the DB were applied in metabolite identification.

Metabolic signaling pathways associated with the differential metabolites were revealed utilizing the MetaboAnalyst5.0 tool (https://www.metaboanalyst.ca/).

### Micro computed tomography (Micro-CT) of mouse lung

The lung tissue density of mice was quantitatively measured by CT, and the degree of pulmonary fibrosis in each group was determined. CT plain scan was performed by the following methods: after anesthesia, the mice were placed on the scanning bed and kept in a supine position, then the chest of the mice was fixed with tape, and the living lung tissue of the mice was scanned after setting the scanning parameters. The images were automatically generated by the system workstation, and the images were post-processed by the relevant system software. The structural and morphological changes of lung tissue were observed to evaluate the different degrees of pulmonary fibrosis with comparison to the control group.

### Quantitative real-time RT-PCR

Total RNA was extracted from mouse lung tissues using RNA-easy Isolation Reagent (Vazyme, Nanjing, China). The concentration and purity of the RNA was determined with a NanoDrop 8000 (Thermo Fisher Scientific, Waltham, MA, USA). cDNA reverse transcription kits (Accurate biology, Hunan, China) were used to reverse transcribe total RNA. The mRNA was quantified by SYBR Green (Thermo Fisher Scientific) on QuantStudio 6 Flex Real-time PCR system (Thermo Fisher Scientific). The *GAPDH* gene was used for normalization. Primers sequences are shown in Supporting Information Table S1. The data were analyzed by 2^–ΔΔCT^ method.

### RNA-seq

The transcriptome sequencing was performed at Magigene Technology Company (Guangzhou, China). mRNA was enriched from total RNA using Oligo(dT)-conjugated magnetic beads and fragmented into short fragments. First-strand cDNA was synthesized using random hexamer primers, followed by second-strand cDNA synthesis. After end repair, A-tailing, and adapter ligation, the cDNA libraries were size-selected using AMPure XP beads (Beckman Coulter, Brea, CA, USA) and amplified by PCR. The qualified libraries were sequenced on an Illumina NovaSeq 6000 platform (Illumina, San Diego, CA, USA) with a PE150 strategy (150 bp paired-end reads). Raw sequencing data (Raw Reads) were filtered using fastp (v0.19.7) to remove adapter sequences and low-quality reads. The parameters were set as follows: qualified quality threshold of 15, sliding window size of 4 with mean quality ≥ 20, and reads length ≥ 75 bp after trimming. Ribosomal RNA sequences were removed by aligning clean reads to the NCBI and Rfam rRNA databases using Bowtie2 (v2.33). The remaining reads were then aligned to the mouse reference genome (mm10) using HISAT2 (v2.1.0). Differential expression analysis was performed with edgeR R package. Functional enrichment analysis of the DEGs was performed using DAVID software (online at http://david.abcc.ncifcrf.gov/). The bioinformatics was plotted by https://www.bioinformatics.com.cn for data visualization.

### Proteomics

The following procedure was performed by Jingjie PTM Biolabs. The sample was grinded with liquid nitrogen into cell powder and then transferred to a 5 mL centrifuge tube. After that, four volumes of lysis buffer (8 M urea, 1% protease inhibitor cocktail) were added to the cell powder, followed by sonication three minutes on ice using a high intensity ultrasonic processor (Scientz). The remaining debris was removed by centrifugation at 12,000 g at 4 °C for 10 min. Finally, the supernatant was collected and the protein concentration was determined with BCA kit according to the manufacturer’s instructions.

The sample was slowly added to the final concentration of 20% (m/v) TCA to precipitate protein, then vortexed to mix and incubated for 2 h at 4 °C. The precipitate was collected by centrifugation at 4500 g for 5 min at 4 °C. The precipitated protein was washed with pre-cooled acetone for 3 times and dried for 1 min. The protein sample was then redissolved in 200 mM TEAB and ultrasonically dispersed. Trypsin was added at 1:50 trypsin-to-protein mass ratio for digestion overnight. The sample was reduced with 5 mM dithiothreitol for 30 min at 56 °C and alkylated with 11 mM iodoacetamide for 15 min at room temperature in darkness. Finally, the peptides were desalted by Strata X SPE column.

To enrich modified peptides, tryptic peptides dissolved in NETN buffer (100 mM NaCl, 1 mM EDTA, 50 mM Tris-HCl, 0.5% NP-40, pH 8.0) were incubated with pre-washed antibody beads at 4 °C overnight with gentle shaking. Then the beads were washed for four times with NETN buffer and twice with H_2_O. The bound 2 peptides were eluted from the beads with 0.1% trifluoroacetic acid. Finally, the eluted fractions were combined and vacuum-dried. For LC-MS/MS analysis, the resulting peptides were desalted with C18 ZipTips (Millipore) according to the manufacturer’s instructions.

The tryptic peptides were dissolved in solvent A, directly loaded onto a home-made reversed-phase analytical column (25-cm length, 100 μm i.d.). The mobile phase consisted of solvent A (0.1% formic acid, 2% acetonitrile in water) and solvent B (0.1% formic acid in acetonitrile). Peptides were separated with following gradient: 0–18 min, 6%-22%B; 18–22 min, 22%-30%B; 22–26 min, 30%-80%B; 26–30 min, 80%B, and all at a constant flow rate of 450 nl/minon a NanoElute UHPLC system (Bruker Daltonics). The peptides were subjected to capillary source followed by the timsTOF Pro 2 mass spectrometry. The electrospray voltage applied was 1.65 kV. Precursors and fragments were analyzed at the TOF detector. The timsTOF Pro was operated in data independent parallel accumulation serial fragmentation (dia-PASEF) mode. The full MS scan was set as100-1700 (MS/MS scan range) and 8PASEF (MS/MS mode) -MS/MS scans were acquired per cycle. The MS/MS scan range was set as 425–1025 and isolation window was set as25 m/z.

### Statistical analyses

Statistical analyses were performed using Graphpad Prism 9 (GraphPad Software, San Diego, CA, USA). Data are presented as mean ± standard error of the mean (SEM). Sample sizes (n) represent the number of independent biological replicates. Statistical analyses were performed with either an unpaired two-sided student’s t-test or one-way ANOVA with Tukey’s post hoctest with *p* < 0.05 considered statistically significant.

## Results

### β-hydroxybutyrylation is significantly decreased in pulmonary fibrosis

To investigate the potential involvement of protein PTMs in pulmonary fibrosis, we first examined lung tissue sections from patients with bronchiolitis obliterans (BO) and healthy controls. Masson’s trichrome staining revealed substantial collagen deposition and fibrotic remodeling in BO tissues (Fig. S1). Immunohistochemical (IHC) analysis of key PTMs—including acetylation (Kac), Kbhb, crotonylation (Kcr), and succinylation (Ksu)—demonstrated significant dysregulation of Kac and Kbhb in fibrotic tissues (Fig. S1). Notably, global Kbhb levels were substantially reduced in fibrotic tissues (Fig. S1).

**Fig. 1:**
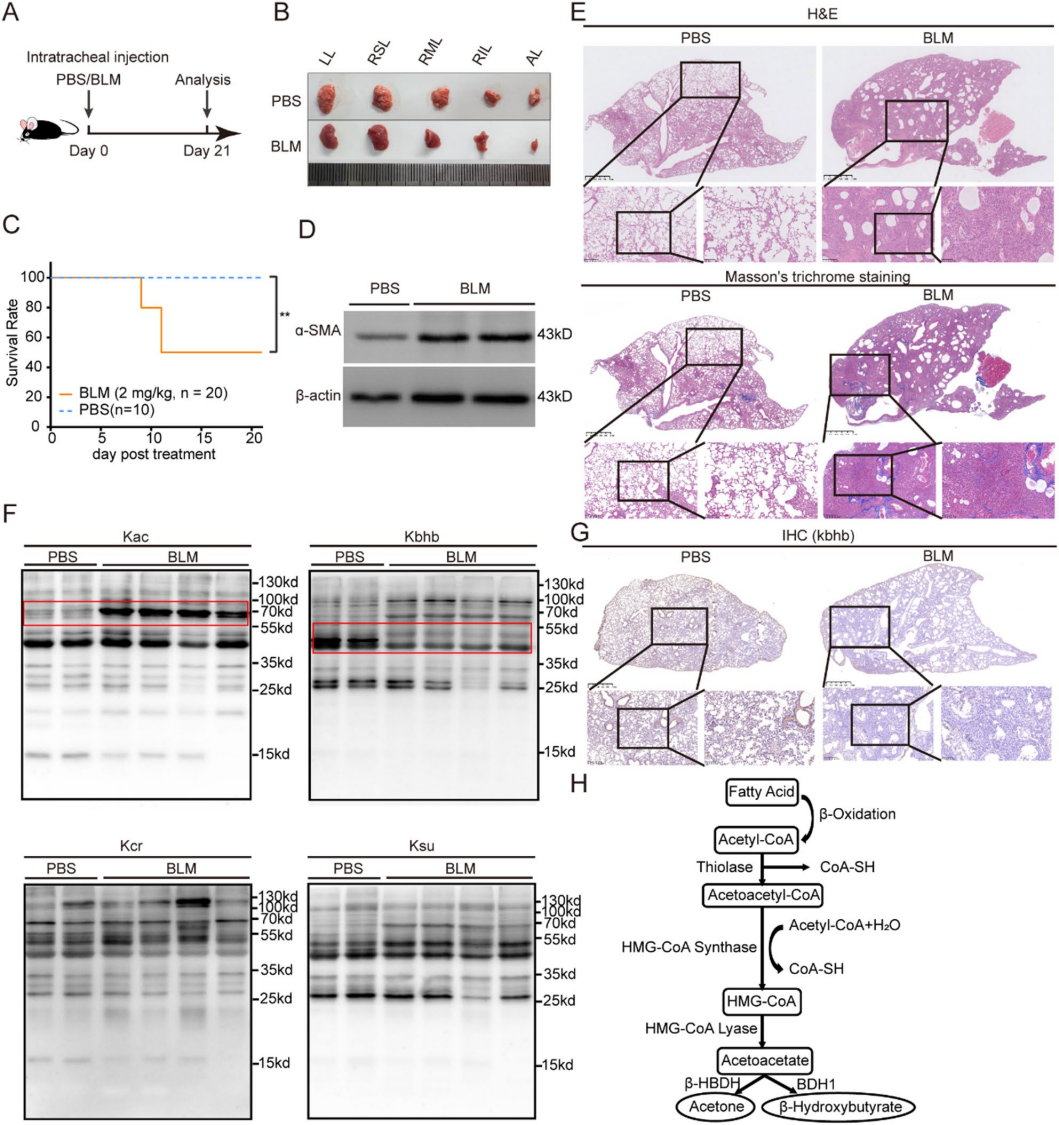
β-hydroxybutyrylation (Kbhb) is significantly decreased in the bleomycin (BLM)-induced murine model of pulmonary fibrosis.** A** Schematic of the BLM-induced pulmonary fibrosis model. C57BL/6 male mice received intratracheal BLM (2 mg/kg) under anesthesia, and lung tissues were harvested for analysis on day 21 post-instillation. **B** Representative images of lung injury in mice 21 days after BLM induction, *n* = 3 (left lobe, LL; right superior lobe, RSL; right middle lobe, RML; right inferior lobe, RIL; accessory lobe, AL). **C** Kaplan–Meier survival curves of PBS (control) and BLM-treated mice, *n* = 10, 20 (*P*-values were from log-rank tests). **D** Western blot analysis of α-SMA expression in PBS (control) and BLM-treated lung tissues, *n* = 3. **E** Representative lung tissue sections from PBS (control) and BLM-treated mice at day 21, stained with H & E (up) and Masson’s trichrome (down), for histopathological assessment, *n* = 6. Scale bars, 1 mm. **F**,** G** Reduced global Kbhb levels in BLM-treated lungs were detected by **(F)** Western blot and **(G)** Immunohistochemistry (IHC). Scale bars, 1 mm. **F** Western blot analysis of key PTMs (Kac: acetylation; Kbhb: β-hydroxybutyrylation; Kcr: crotonylation; Ksu: succinylation), *n* = 2, 4. **G** Kbhb were decreased in BLM-treated lungs as shown by immunohistochemical analysis, *n* = 6. **H** Schematic diagram of the ketone body metabolism pathway, highlighting β-hydroxybutyrate production via hepatic fatty acid β-oxidation. All experimental data (**B**-**H**) were verified in at least three independent experiments. **P < 0.01

To further corroborate these observations, we employed a BLM-induced murine model of pulmonary fibrosis. Mice were euthanized on day 21 following BLM administration (Fig. 1A), as previous studies have demonstrated that this time point represents the established fibrotic phase characterized by significant collagen deposition and alveolar structural remodeling in C57BL/6 mice [17, 18]. Widespread lung injury was observed across all pulmonary lobes in the BLM group (Fig. 1B), accompanied by a significant reduction in survival rate (Fig. 1C). We further detected that BLM-treated mice induced protein expression of α-smooth muscle actin (α-SMA), which is associated with pulmonary fibrosis (Fig. 1D). Using H&E staining and Masson’s trichrome staining on lung tissue sections, we observed that BLM treatment promoted collagen deposition and increased fibrotic area in mice (Fig. 1E). Most importantly, and consistent with the human data, both western blot and immunohistochemistry confirmed a marked decrease in global Kbhb levels in BLM-treated mouse lungs (Fig. 1F and G).

These results demonstrate a marked reduction in Kbhb in both human and experimental pulmonary fibrosis, suggesting a potential role for Kbhb in fibrogenesis. Notably, the metabolic precursor for Kbhb, β-OHB, is produced during hepatic ketogenesis via fatty acid β-oxidation (Fig. 1H). This pathway shares acetyl-CoA as a common precursor with protein acetylation, pointing to possible crosstalk between ketone body metabolism and PTM regulation in fibrosis.

### β-hydroxybutyrylcarnitine is significantly reduced in lung tissues of BLM model mice

**Fig. 2:**
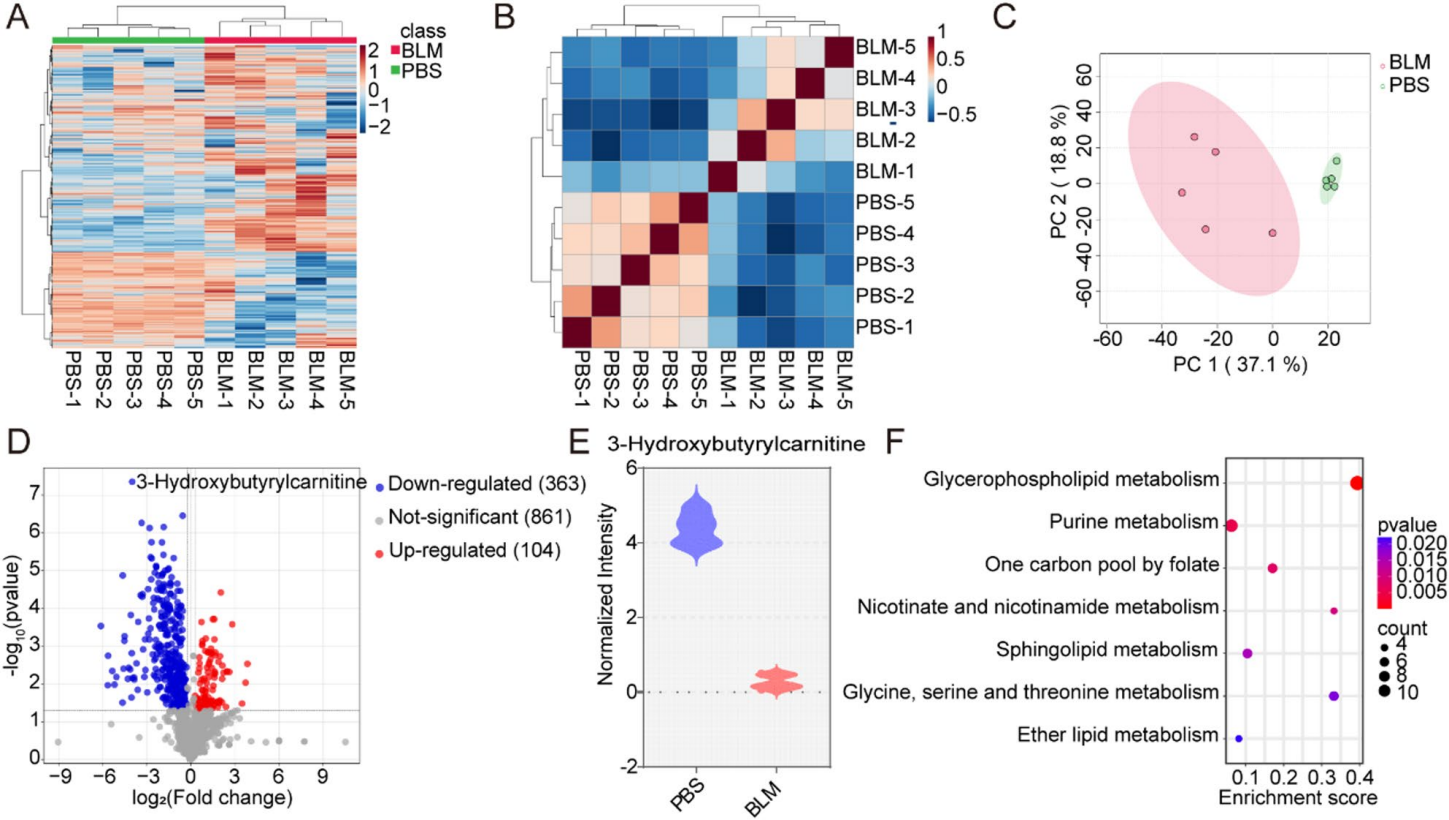
Metabolomics identifies β-hydroxybutyrylcarnitine reduction in BLM-induced model mice.** A** Heatmap analysis of metabolomics profiles in mouse lung tissues. Rows represent individual metabolites (clustered by expression patterns), and columns represent samples from PBS-treated control (PBS) and bleomycin-induced fibrosis groups (BLM). Color scale indicates normalized metabolite expression levels (blue: downregulation; red: upregulation). **B** Correlation analysis of metabolomics profiles in pulmonary fibrosis. The heatmap depicts correlation coefficients (r) between individual samples from BLM and PBS groups. Color intensity represents the degree of correlation (red: positive; blue: negative). **C** Plot of Principal Component Analysis (PCA) of samples from the same cohort of samples in the PBS (control) and BLM (disease) groups. **D** Differential metabolite screening volcano map (|log_2_FoldChange (FC)| > 1, FDR < 0.05). **E** Violin plot showing that β-hydroxybutyrylcarnitine abundance was significantly reduced in the BLM group compared to the PBS control group. **F** KEGG pathway enrichment analysis of differentially abundant metabolites. In (**A-F**), *n* = 5 biologically independent samples. *P*-values were obtained using unpaired two-tailed Student’s t-test

To investigate pulmonary metabolic alterations in the BLM model, we performed untargeted metabolomic analysis. Hierarchical clustering revealed distinct metabolic signatures between fibrotic and control lungs, highlighting potential dysregulated pathways in pulmonary fibrosis (Fig. 2A). Furthermore, correlation analysis revealed strong intra-group metabolic homogeneity in fibrotic lungs (BLM) and distinct metabolic divergence between BLM and control groups (Fig. 2B). Collectively, the data provide clear evidence of systemic metabolic reprogramming in pulmonary fibrosis.

Both PCA and OPLS-DA models revealed distinct clustering, effectively separating samples from BLM and control groups (Fig. 2C and Fig. S2A). Among the altered metabolites, β-hydroxybutyrylcarnitine levels were significantly reduced in BLM-exposed lungs compared to controls (Fig. 2D). This metabolite, an intermediate in fatty acid β-oxidation linked to both fatty acid and ketone body metabolism [19], ranked among the top 15 metabolites in the VIP analysis, underscoring its potential biological significance (Fig. S2B). In addition, direct analysis of the expression level of β-hydroxybutyrylcarnitine confirmed its significant decrease in the BLM group (Fig. 2E). Pathway enrichment analysis (KEGG) of differential metabolites revealed significant associations with glycerophospholipid metabolism, purine metabolism, and one-carbon pool by folate, among others (Fig. 2F). Consistently, genes involved in glycerophospholipid and purine metabolism formed distinct clusters in the heatmap analysis (Fig. S2C). Collectively, these results demonstrate a significant reduction of β-hydroxybutyrylcarnitine in the lungs of BLM model mice.

### Restoration of β-hydroxybutyrate attenuates pulmonary fibrosis in vivo

**Fig. 3:**
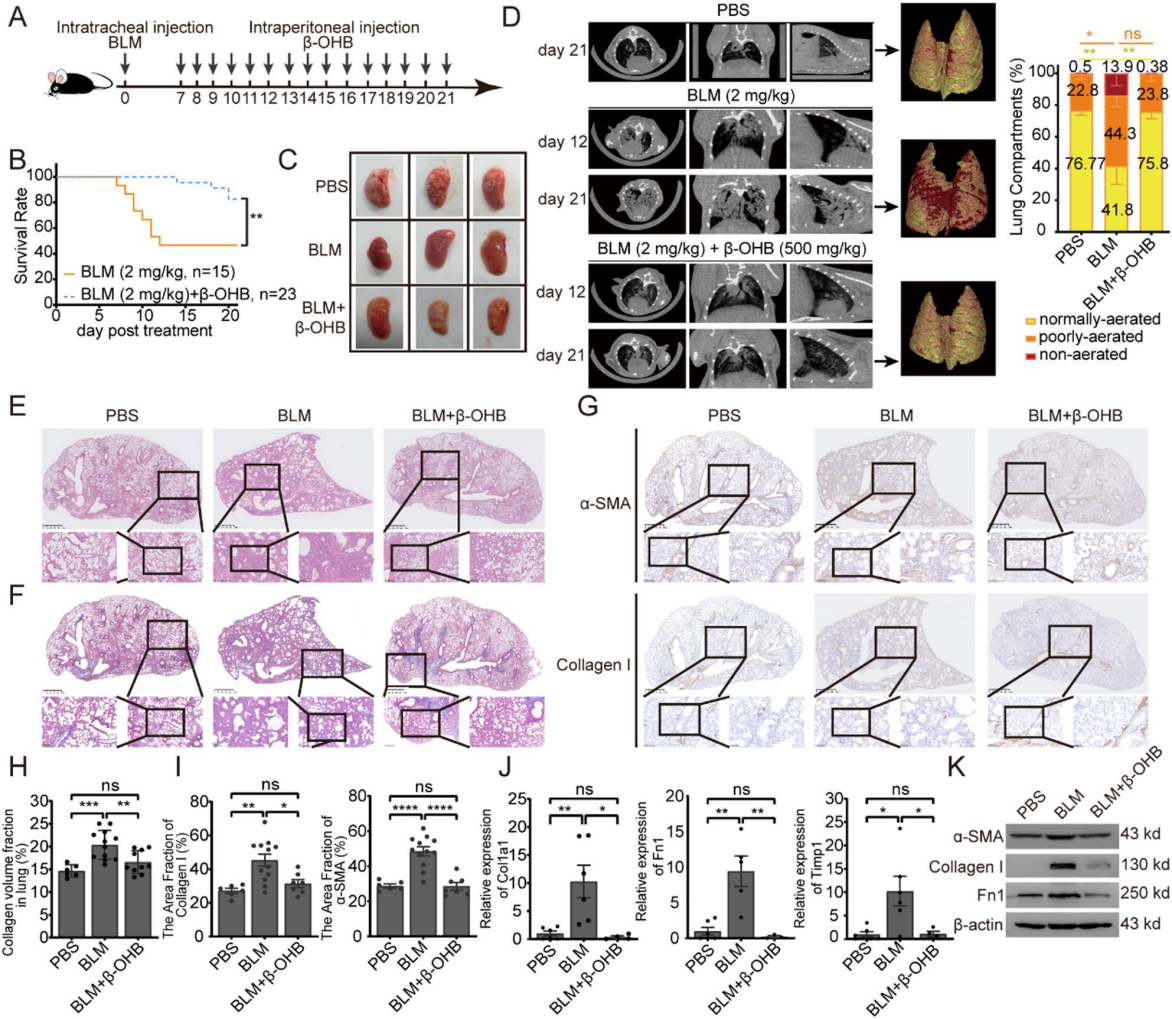
β-Hydroxybutyrate (β-OHB) supplementation attenuates pulmonary fibrosis progression.** A** Procedure for the establishment of the β-Hydroxybutyrate (β-OHB) repletion mouse model. C57BL/6 male mice were administered daily intraperitoneal injections of β-OHB from day 7 to day 21 after intratracheal BLM-induced pulmonary fibrosis. Lung tissues were harvested on day 21. **B** Kaplan–Meier survival curves of BLM-induced mice with or without β-OHB treatment, *n* = 15, 23. (*P*-values were from log-rank tests). **C** Representative images of lung injury in BLM-induced mice with or without β-OHB, *n* = 3. **D** Micro-CT analysis of lung structure and function in BLM-induced mice with or without β-OHB, *n* = 6. **E**,** F** Histopathological analysis of H & E Staining (**E**) and Masson’s trichrome staining (**F**). **E** Representative lung tissue sections from PBS (control), BLM-treated and BLM + β-OHB-treated mice at day 21, stained with H & E Staining, *n* = 6. **F** Representative lung tissue sections from PBS (control), BLM-treated and BLM + β-OHB-treated mice at day 21, stained with Masson’s trichrome staining, *n* = 6. **G** Immunohistochemical experiments detected lighter staining for α-SMA and Collagen I in β-OHB treated BLM-mice, *n* = 6. **H** Quantification of collagen volume fraction from Masson’s trichrome staining. **I** Quantification of ɑ-SMA and collagen I positive areas by immunohistochemistry. **J** qPCR analysis of the fibrosis-related genes in BLM-induced mice with or without β-OHB; **K** Western blot of fibrosis markers in BLM-induced mice with or without β-OHB. In **J-K**, at least three biologically independent samples. In **H-J**, data are shown as the mean ± SEM. *P*-values were obtained using one-way ANOVA with Tukey’s post hoctest. All experimental data were verified in at least three independent experiments. *P < 0.05, **P < 0.01, ***P < 0.001, ****P < 0.0001

To investigate the therapeutic potential of enhancing Kbhb, we administered β-hydroxybutyrate (β-OHB) via daily intraperitoneal injection from day 7 to day 21 after BLM induction (Fig. 3A). β-OHB supplementation significantly improved the survival rate of BLM-treated mice (Fig. 3B), and markedly ameliorated BLM-induced lung injury (Fig. 3C). Micro-computed tomography (micro-CT) imaging revealed that β-OHB treatment significantly attenuated BLM-induced structural lung damage and markedly improved pulmonary ventilation (Fig. 3D). Using H&E staining and Masson’s trichrome staining on lung tissue sections, we observed that β-OHB administration reduced lung architectural distortion and collagen deposition (Fig. 3E, F, H). Immunohistochemical analysis indicated decreased expression of fibrotic markers α-SMA and Collagen I in β-OHB-treated groups (Fig. 3G and I). Additionally, qPCR and western blot analyses confirmed the downregulation of fibrosis-related genes at both transcriptional and protein levels after β-OHB intervention (Fig. 3J and K). These findings collectively indicate that restoration of β-hydroxybutyrate levels confers protective effects against BLM-induced pulmonary fibrosis by improving survival outcomes and attenuating lung injury.

### β-hydroxybutyrylation regulates pulmonary fibrosis through the focal adhesion and PI3K/AKT pathway

**Fig. 4:**
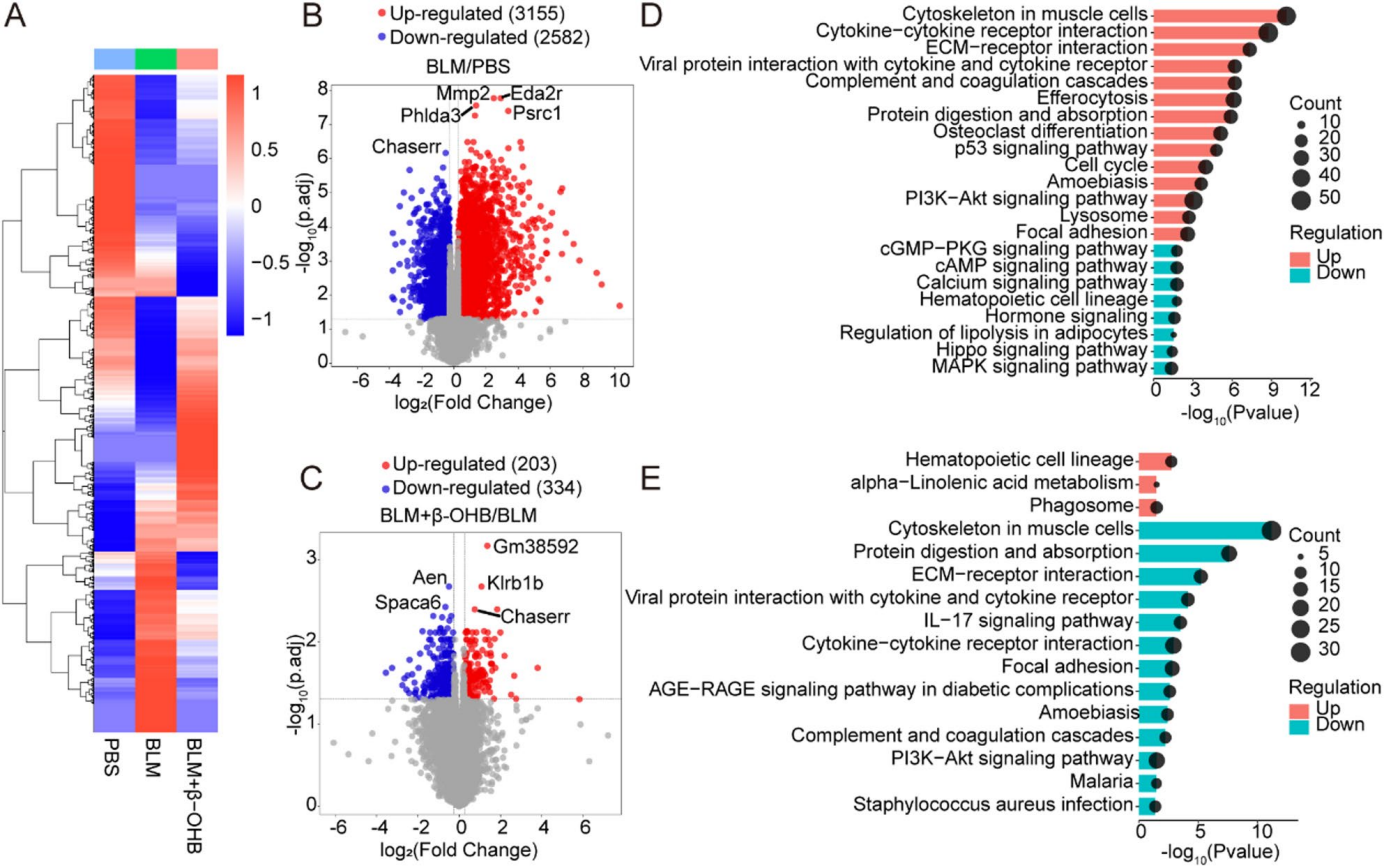
Transcriptome analysis demonstrating the involvement of focal adhesion (FA) pathway and PI3K/AKT pathway in the regulation of pulmonary fibrosis by β-hydroxybutyrylation. **A** Heatmap displays normalized gene expression values (Z-scores) in bleomycin-induced fibrosis (BLM), β-hydroxybutyrate-treated fibrosis (BLM + β-OHB), and control (PBS) groups. **B**,** C** Volcano plots of differentially expressed genes (|log_2_FC| > 1, FDR < 0.05) in BLM/PBS (**B**) and BLM + β-OHB/BLM (**C**). **D**,** E** Screening of the obtained differential gene KEGG pathway enrichment analysis in BLM/PBS (**D**) and BLM + β-OHB/BLM (**E**), showing that both the FA pathway and the PI3K/AKT pathway were significantly enriched after restoring of β-OHB. In **A-E**, *n* = 6 (PBS, BLM) or 7 (BLM + β-OHB) biologically independent samples

To further investigate the role of β-OHB in pulmonary fibrosis, we performed transcriptome sequencing. The results demonstrated that β-OHB intervention significantly attenuated BLM-induced transcriptional alterations, as evidenced by the intermediate gene expression profile of the BLM + β-OHB group relative to BLM and PBS groups (Fig. 4A). Moreover, Transcriptome analysis of mouse lung tissues revealed significant differences between the PBS, BLM and β-hydroxybutyrate restoration (BLM + β-OHB) groups (Fig. 4B and C). KEGG enrichment analysis of differentially expressed genes showed significant upregulation of the focal adhesion (FA) and PI3K/AKT pathways in the BLM group compared to the PBS group. Importantly, β-hydroxybutyrate restoration significantly downregulated both pathways in the BLM + β-OHB group (Fig. 4D and E). These findings suggest that Kbhb modulates pulmonary fibrosis via the FA and PI3K/AKT signaling pathways.

### β-hydroxybutyrate exerts anti-fibrotic effects by directly regulating Zyxin

**Fig. 5:**
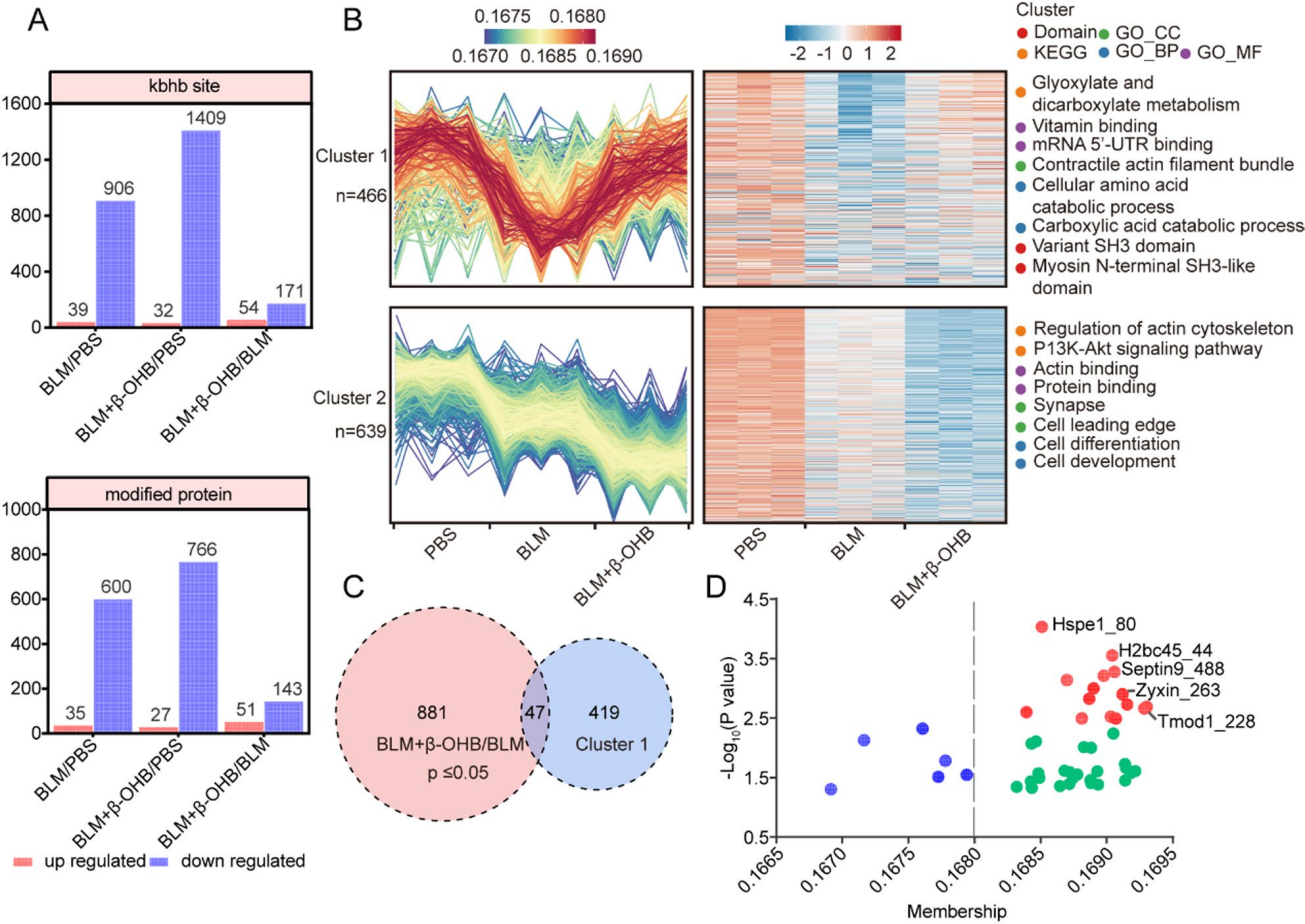
Proteomic profiling reveals Zyxin as a key β-hydroxybutyrylation (Kbhb) target in pulmonary fibrosis.** A** Differential analysis of β-hydroxybutyrylated modifier proteins and modification sites in BLM/PBS, BLM + β-OHB/PBS and BLM + β-OHB/BLM groups (|log_2_FC| > 0.585, *P* < 0.05). **B** Trend analysis of protein β-hydroxybutyrylation (Kbhb) levels identified 466 significantly altered targets that were significantly down-regulated in BLM/PBS and significantly up-regulated in BLM + β-OHB/BLM. **C** Venn diagram identifying 47 consensus proteins meeting both differential and trend criteria. **D** Membership scores reflect the confidence of a protein’s assignment to the dominant trend cluster, with higher scores indicating a stronger association. In (A-D), *n* = 3 biologically independent samples. *P*-values were obtained using unpaired two-tailed Student’s t-test

To identify proteins targeted by Kbhb in IPF, we performed proteomic analysis of this modification in mouse lungs across three groups: the PBS control, BLM-induced IPF disease and β-hydroxybutyrate restoration (BLM + β-OHB) groups. The proteomic analysis revealed significant alterations in Kbhb profiles (Fig. 5A). Trend analysis identified 466 proteins significantly downregulated by Kbhb in the BLM group and upregulated in the BLM + β-OHB group (Fig. 5B). Intersecting these with differentially expressed proteins yields 47 candidates (Fig. 5C). Further filtering by membership trend scores (Fig. 5D) identified Zyxin as a key target protein of Kbhb.

### Zyxin-knockdown attenuates pulmonary fibrosis in mice

**Fig. 6:**
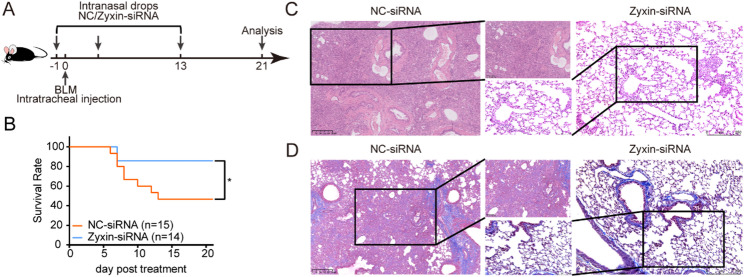
Zyxin knockdown significantly reduces lung fibrosis and decreases mortality in mice, demonstrating that Zyxin has an important role in pulmonary fibrosis.** A** The process of establishing the Zyxin-knockdown model mouse. Beginning one day before intratracheal BLM induction in C57BL/6 male mice, si-Zyxin was administered intranasally every 7 days (with a total of three administrations). Lung tissues were harvested for analysis on day 21 post-BLM induction. **B** Kaplan–Meier survival curves of Zyxin-knockdown model mice, *n* = 14, 15 (*P*-values were from log-rank tests). **C**,** D** H & E (**C**) and Masson’s trichrome staining (**D**). **C** Representative H & E-stained lung sections are shown for the control and Zyxin-knockdown group at day 21 after BLM induction, *n* = 6. **D** Representative Masson’s trichrome-stained lung sections are shown for the control and Zyxin-knockdown group at day 21 after BLM induction, *n* = 6. **P < 0.01

To investigate the functional contribution of Zyxin to pulmonary fibrogenesis, we performed lung-specific knockdown of Zyxin through intranasal administration of Zyxin-targeting siRNA following BLM challenge (Fig. 6A). The efficiency of Zyxin knockdown was confirmed by quantitative PCR, which showed significant reduction of Zyxin mRNA expression in lung tissues of siRNA-treated mice (Fig. S4A). Zyxin knockdown significantly improved survival rates in BLM-induced mice compared to the negative control group (Fig. 6B). Histopathological evaluation using H&E and Masson’s trichrome staining demonstrated that Zyxin depletion markedly attenuated BLM-induced collagen deposition and architectural distortion (Fig. 6C and D). Furthermore, in vitro validation in mouse lung fibroblast (MLg) cells demonstrated that transfection with Zyxin-specific siRNA (si-Zyxin) significantly reduced Zyxin mRNA levels compared to negative control (si-NC) (Fig. S4B). Notably, baseline expression analysis revealed that Zyxin is predominantly expressed in lung fibroblasts rather than macrophages (Fig. S4C), suggesting its specific role in fibroblast-mediated fibrotic processes. Collectively, these findings identify Zyxin as a novel target of Kbhb, a post-translational modification whose impact on fibrosis progression suggests the therapeutic potential for targeting this pathway.

## Discussion

IPF remains a devastating disease with limited therapeutic options, underscoring the critical need to elucidate its underlying molecular mechanisms. While dysregulation of PTMs such as acetylation and ubiquitination has been implicated in IPF pathogenesis [12, 20], the role of many other modifications, particularly those linked to cellular metabolism, remains largely uncharted. Previous studies have found that IPF lungs exhibit upregulated type I and type II histone deacetylase (HDAC), with increased histone acetylation promoting fibrotic gene expression [10]. Additionally, Spry1 ubiquitination in lung fibroblasts activates Ras-ERK-p70S6K pathway and promotes myofibroblast differentiation [12]. However, roles for other PTMs in IPF remains unexplored. Our study unveils a previously unrecognized nexus between metabolic reprogramming and fibrotic signaling by identifying a significant reduction in global β-hydroxybutyrylation (Kbhb) in fibrotic lungs from both human patients and BLM-induced mouse models. This central observation positions Kbhb not merely as a bystander but as a potential key regulator in pulmonary fibrogenesis.

β-hydroxybutyrylation is mediated by β-OHB, a metabolite implicated in development [21], memory T cell maintenance [22], and multiple pathologies including cardiovascular [23], neurological [24], renal [25], metabolic [26], and oncological [27] diseases. However, there is a lack of studies on the regulation of IPF by Kbhb. The functional significance of this finding is robustly demonstrated by our interventional approach. Administration of β-OHB, the direct precursor for Kbhb, successfully restored global Kbhb levels and, more importantly, conferred remarkable therapeutic benefits—attenuating histopathological damage, reducing collagen deposition, and significantly improving survival. This evidence moves beyond correlation to establish a causal link between the loss of Kbhb and disease progression, suggesting that targeting this pathway holds substantial therapeutic promise.

Furthermore, Kbhb modifies both histones that regulate transcriptional activation and non-histone proteins that modify protein activity [13, 28]. Histone Kbhb is associated with starvation-associated gene activation [13], promotes VEGF production to attenuate vascular damage [23] and facilitates BDNF synthesis in neurological contexts [24]. In tumor cells, P53 kbhb modification competitively inhibits P53 acetylation, dampening its transcriptional activity [29]. These studies collectively underscore the capacity of Kbhb to orchestrate broad transcriptional reprogramming, which aligns with our transcriptomic data showing that β-OHB normalizes the dysregulated gene expression profile in fibrotic lungs, particularly through the focal adhesion and PI3K/AKT pathways.

Meanwhile, Zyxin has been shown to be involved in the formation of myocardial [30] and dermal fibrosis [16]. Huang et al. [16] demonstrated that Zyxin inhibition significantly alleviated bleomycin-induced skin fibrosis in mice, with effects observed on day 21 post-induction, and further revealed that Zyxin regulates fibrosis through the FAK/PI3K/AKT and TGF-β signaling pathways. Consistent with these findings, our study demonstrates that lung-specific Zyxin knockdown significantly reduces pulmonary fibrosis and improves survival in mice (Fig. 6).

How might Zyxin Kbhb orchestrate its anti-fibrotic effects? Our multi-omics data offer compelling clues. Proteomics identified Zyxin K263 as a key β-hydroxybutyrylation (Kbhb) site, which was significantly reduced in fibrotic lung tissues and restored upon β-OHB supplementation (Fig. 5). Transcriptomic analysis further revealed that β-OHB treatment significantly downregulated the PI3K/AKT signaling pathway in fibrotic lungs (Fig. 4D and E). The PI3K/AKT pathway is a key driver of IPF pathogenesis. Its inhibition reduces morbidity and mortality [31]. Studies have shown that the PI3K/AKT pathway promotes fibroblast differentiation, collagen secretion and M2-type macrophage polarization [32], which in turn mediate the secretion of pro-fibrotic factors such as TGF-β, IL-4, and IL-13 [33–35].The activation of AKT requires the dual phosphorylation of both the T308 and S473 sites. Recently, it was found that in tumor cells, acetylation of lysine residues (K14 and K20) at positions 14 and 20 of AKT affects its binding to PIP3 and thus AKT activation.

Based on these observations, we propose that β-hydroxybutyrylation of Zyxin at K263 acts as a molecular switch: under normal conditions, Zyxin Kbhb inhibits PI3K/AKT pathway activation, thereby blocking fibroblast activation and collagen deposition; in the fibrotic state, reduced Kbhb modification at Zyxin K263 leads to diminished inhibition of the PI3K/AKT pathway, resulting in aberrant pathway activation that promotes pulmonary fibrosis. β-OHB supplementation restores Zyxin K263 Kbhb levels, re-inhibits PI3K/AKT signaling, and attenuates fibrotic progression. This model establishes a direct molecular link between ketone body metabolism (β-OHB), post-translational modification (Kbhb), and fibrotic signaling (PI3K/AKT), offering a novel therapeutic paradigm for IPF. While the exact mechanism—whether through altering Zyxin’s interactome, its subcellular localization, or its stability—requires further dissection, our findings provide a solid foundation for this novel regulatory paradigm.

While our findings provide compelling evidence for the role of Zyxin Kbhb in attenuating pulmonary fibrosis, several limitations of this study should be acknowledged. First, while our human samples demonstrated a marked reduction in Kbhb levels in fibrotic lung tissues, detailed clinical information regarding the exact duration of fibrotic disease was not available due to the retrospective nature of tissue collection. Prospective studies with well-documented longitudinal clinical data are needed to further elucidate the temporal relationship between Kbhb reduction and fibrotic progression. Second, the precise molecular mechanism by which Zyxin Kbhb inhibits the PI3K/AKT pathway remains to be fully elucidated. Although our transcriptomic data strongly associate β-OHB treatment with downregulation of this pathway, the direct molecular link—whether Zyxin Kbhb modulates PI3K/AKT signaling through altered protein-protein interactions at focal adhesion sites, or through its nuclear shuttling capability to regulate gene expression—requires further investigation. Notwithstanding these limitations, our study unequivocally identifies Zyxin Kbhb as a critical novel regulator of pulmonary fibrosis. We demonstrate that the restoration of this modification, via a readily available metabolic substrate, offers a potent therapeutic strategy. These findings not only deepen our understanding of the metabolic regulation of fibrotic diseases but also pave the way for novel, mechanism-based interventions for IPF.

## Supplementary Information

Supplementary Material 1.

Supplementary Material 2.

## Data Availability

The datasets generated and analyzed during the current study are available in public repositories under the following accession numbers: The raw RNA-seq transcriptomic data have been deposited in the Genome Sequence Archive (GSA) of the China National Center for Bioinformation (CNCB) under BioProject accession number PRJCA053555.The metabolomics data have been deposited in the MetaboLights database under accession number MTBLS13585.The proteomics data have been deposited in the ProteomeXchange Consortium via the PRIDE partner repository under dataset identifier PXD072987.These data are publicly accessible via the respective repository websites.

## References

[1] Zhao M, Wang L, Wang M, Zhou S, Lu Y, Cui H et al. Targeting fibrosis: mechanisms and clinical trials. Signal Transduct Target Therapy. 2022;7(1):206. 10.1038/s41392-022-01070-3 PMC9247101 35773269

[2] Lederer DJ, Longo DL, Martinez FJ. Idiopathic Pulmonary Fibrosis. N Engl J Med. 2018;378(19):1811–23. 29742380 10.1056/NEJMra1705751

[3] Moss BJ, Ryter SW, Rosas IO. Pathogenic Mechanisms Underlying Idiopathic Pulmonary Fibrosis. Annu Rev Pathol. 2022;17(1):515–46. 34813355 10.1146/annurev-pathol-042320-030240

[4] Shu F, Xiao H, Li Q-N, Ren X-S, Liu Z-G, Hu B-W et al. Epigenetic and post-translational modifications in autophagy: biological functions and therapeutic targets. Signal Transduct Target Therapy. 2023;8(1):32. 10.1038/s41392-022-01300-8 PMC9842768 36646695

[5] Lee JM, Hammarén HM, Savitski MM, Baek SH. Control of protein stability by post-translational modifications. Nat Commun. 2023;14(1):201. 10.1038/s41467-023-35795-8 PMC9839724 36639369

[6] Wu X, Xu M, Geng M, Chen S, Little PJ, Xu S et al. Targeting protein modifications in metabolic diseases: molecular mechanisms and targeted therapies. Signal Transduct Target Therapy. 2023;8(1):220. 10.1038/s41392-023-01439-y PMC10224996 37244925

[7] Geffen Y, Anand S, Akiyama Y, Yaron TM, Song Y, Johnson JL, et al. Pan-cancer analysis of post-translational modifications reveals shared patterns of protein regulation. Cell. 2023;186(18):3945–e6726. 37582358 10.1016/j.cell.2023.07.013 PMC10680287

[8] Jiang W, Wang M, Wang J, Hao Q, Li Y, Liu L et al. β-Hydroxybutyrate promotes cancer metastasis through β-hydroxybutyrylation-dependent stabilization of Snail. Nat Commun. 2025;16(1):6592. 10.1038/s41467-025-61541-3 PMC12271565 40675949

[9] Lin Z, Zhao S, Li X, Miao Z, Cao J, Chen Y, et al. Cathepsin B S-nitrosylation promotes ADAR1-mediated editing of its own mRNA transcript via an ADD1/MATR3 regulatory axis. Cell Res. 2023;33(7):546–61. 37156877 10.1038/s41422-023-00812-4 PMC10313700

[10] Zhang X, Liu H, Zhou JQ, Krick S, Barnes JW, Thannickal VJ, et al. Modulation of H4K16Ac levels reduces pro-fibrotic gene expression and mitigates lung fibrosis in aged mice. Theranostics. 2022;12(2):530–41. 34976199 10.7150/thno.62760 PMC8692895

[11] Liu S-s, Lv X-x, Liu C, Qi J, Li Y-x, Wei X-p, et al. Targeting Degradation of the Transcription Factor C/EBPβ Reduces Lung Fibrosis by Restoring Activity of the Ubiquitin-Editing Enzyme A20 in Macrophages. Immunity. 2019;51(3):522–e347. 31471107 10.1016/j.immuni.2019.06.014

[12] Liu S-s, Liu C, Lv X-x, Cui B, Yan J, Li Y-x, et al. The chemokine CCL1 triggers an AMFR-SPRY1 pathway that promotes differentiation of lung fibroblasts into myofibroblasts and drives pulmonary fibrosis. Immunity. 2021;54(9):2042–e568. 34407391 10.1016/j.immuni.2021.06.008

[13] Xie Z, Zhang D, Chung D, Tang Z, Huang H, Dai L, et al. Metabolic Regulation of Gene Expression by Histone Lysine β-Hydroxybutyrylation. Mol Cell. 2016;62(2):194–206. 27105115 10.1016/j.molcel.2016.03.036 PMC5540445

[14] DA N. MC B. Nuclear-cytoplasmic shuttling of the focal contact protein, zyxin: a potential mechanism for communication between sites of cell adhesion and the nucleus. J Cell Biol. 1996;138(5):1139–47. 10.1083/jcb.138.5.1139 PMC2136768 9281590

[15] Kotb A, Hyndman ME, Patel TR. The role of zyxin in regulation of malignancies. Heliyon. 2018;4(7):e00695. 10.1016/j.heliyon.2018.e00695 PMC6072900 30094365

[16] Huang Y, Zhao H, Zhang Y, Tang Y, Shi X, Jiang S, et al. Enhancement of Zyxin Promotes Skin Fibrosis by Regulating FAK/PI3K/AKT and TGF-β Signaling Pathways via Integrins. Int J Biol Sci. 2023;19(8):2394–408. 37215989 10.7150/ijbs.77649 PMC10197900

[17] Elewa YHA, Ichii O, Takada K, Nakamura T, Masum MA, Kon Y. Histopathological Correlations between Mediastinal Fat-Associated Lymphoid Clusters and the Development of Lung Inflammation and Fibrosis following Bleomycin Administration in Mice. Front Immunol. 2018;9:271. 29497425 10.3389/fimmu.2018.00271 PMC5818413

[18] Gao S, Li X, Jiang Q, Liang Q, Zhang F, Li S, et al. PKM2 promotes pulmonary fibrosis by stabilizing TGF-β1 receptor I and enhancing TGF-β1 signaling. Sci Adv. 2022;8(38):eabo0987. 36129984 10.1126/sciadv.abo0987 PMC9491720

[19] Soeters MR, Serlie MJ, Sauerwein HP, Duran M, Ruiter JP, Kulik W, et al. Characterization of D-3-hydroxybutyrylcarnitine (ketocarnitine): an identified ketosis-induced metabolite. Metabolism. 2012;61(7):966–73. 22209095 10.1016/j.metabol.2011.11.009

[20] Wu J, Gong L, Li Y, Liu T, Sun R, Jia K, et al. SGK1 aggravates idiopathic pulmonary fibrosis by triggering H3k27ac-mediated macrophage reprogramming and disturbing immune homeostasis. Int J Biol Sci. 2024;20(3):968–86. 38250161 10.7150/ijbs.90808 PMC10797695

[21] Chong D, Gu Y, Zhang T, Xu Y, Bu D, Chen Z et al. Neonatal ketone body elevation regulates postnatal heart development by promoting cardiomyocyte mitochondrial maturation and metabolic reprogramming. Cell Discovery. 2022;8(1):106. 10.1038/s41421-022-00447-6 PMC9553951 36220812

[22] Zhang H, Tang K, Ma J, Zhou L, Liu J, Zeng L, et al. Ketogenesis-generated β-hydroxybutyrate is an epigenetic regulator of CD8 + T-cell memory development. Nat Cell Biol. 2019;22(1):18–25. 31871320 10.1038/s41556-019-0440-0

[23] Li B, Yu Y, Liu K, Zhang Y, Geng Q, Zhang F, et al. β-Hydroxybutyrate inhibits histone deacetylase 3 to promote claudin-5 generation and attenuate cardiac microvascular hyperpermeability in diabetes. Diabetologia. 2020;64(1):226–39. 33106900 10.1007/s00125-020-05305-2

[24] Gersner R, Gal R, Levit O, Moshe H, Zangen A. Inherited behaviors, BDNF expression and response to treatment in a novel multifactorial rat model for depression. Int J Neuropsychopharmacol. 2014;17(06):945–55. 24513109 10.1017/S1461145714000030

[25] Torres JA, Kruger SL, Broderick C, Amarlkhagva T, Agrawal S, Dodam JR, et al. Ketosis Ameliorates Renal Cyst Growth in Polycystic Kidney Disease. Cell Metabol. 2019;30(6):1007–e235. 10.1016/j.cmet.2019.09.012 PMC6904245 31631001

[26] Koronowski KB, Greco CM, Huang H, Kim J-K, Fribourgh JL, Crosby P et al. Ketogenesis impact on liver metabolism revealed by proteomics of lysine β-hydroxybutyrylation. Cell Rep. 2021;36(5):109487. 10.1016/j.celrep.2021.109487 PMC8372761 34348140

[27] Zhang H, Chang Z, Qin L-n, Liang B, Han J-x, Qiao K-l et al. MTA2 triggered R-loop trans-regulates BDH1-mediated β-hydroxybutyrylation and potentiates propagation of hepatocellular carcinoma stem cells. Signal Transduct Target Therapy. 2021;6(1):135. 10.1038/s41392-021-00464-z PMC8016859 33795651

[28] Kovács Z, Brunner B, Ari C. Beneficial Effects of Exogenous Ketogenic Supplements on Aging Processes and Age-Related Neurodegenerative Diseases. Nutrients. 2021;13(7):2197. 10.3390/nu13072197 PMC8308443 34206738

[29] Liu K, Li F, Sun Q, Lin N, Han H, You K et al. p53 β-hydroxybutyrylation attenuates p53 activity. Cell Death Dis. 2019;10(3):243. 10.1038/s41419-019-1463-y PMC6411878 30858356

[30] Al-Hasani J, Sens-Albert C, Ghosh S, Trogisch FA, Nahar T, Friede PAP et al. Zyxin protects from hypertension-induced cardiac dysfunction. Cell Mol Life Sci. 2022;79(2):93. 10.1007/s00018-022-04133-4 PMC8786748 35075545

[31] Wang J, Hu K, Cai X, Yang B, He Q, Wang J, et al. Targeting PI3K/AKT signaling for treatment of idiopathic pulmonary fibrosis. Acta Pharm Sinica B. 2022;12(1):18–32. 10.1016/j.apsb.2021.07.023 PMC8799876 35127370

[32] YX W, DY W, YC G. Zyxin: a mechanotransductor to regulate gene expression. Eur Rev Med Pharmacol Sci. 2019;23(1):413–25. 30657586 10.26355/eurrev_201901_16790

[33] Wang Y, Zhang L, Wu G-R, Zhou Q, Yue H, Rao L-Z et al. MBD2 serves as a viable target against pulmonary fibrosis by inhibiting macrophage M2 program. Sci Adv. 2021;7(1):eabb6075. 10.1126/sciadv.abb6075 PMC7775789 33277324

[34] Nie Y, Hu Y, Yu K, Zhang D, Shi Y, Li Y, et al. Akt1 regulates pulmonary fibrosis via modulating IL-13 expression in macrophages. Innate Immun. 2019;25(7):451–61. 31299858 10.1177/1753425919861774 PMC6900639

[35] Yan L, Hou C, Liu J, Wang Y, Zeng C, Yu J, et al. Local administration of liposomal-based Plekhf1 gene therapy attenuates pulmonary fibrosis by modulating macrophage polarization. Sci China Life Sci. 2023;66(11):2571–86. 37340175 10.1007/s11427-022-2314-8

