# Supplementary material for: The β-hydroxybutyrylation of Zyxin ameliorates pulmonary fibrosis by inhibiting lung fibroblast activation through the PI3K/AKT pathway
Source: Respir Res. 2026 May 30;27:308. doi: 10.1186/s12931-026-03732-0 (PMC13479915; doi:10.1186/s12931-026-03732-0)
Supplement: Supplementary file 1 [file 12931_2026_3732_MOESM1_ESM.docx]

**Supporting tables**

**Table S1** The siRNA sequences.

| **siRNA** | **Sense** | **Antisense** |
| --- | --- | --- |
| si-*Zyxin* | 5′-GCUGAUGCAGGACAUGGAATT-3′ | 5′-TTCGACUACGUCCUGUACCUU-3′ |

**Table S2** The primers sequences.

| **Gene** | **Forward primer** | **Reverse primer** |
| --- | --- | --- |
| Mus *Gapdh* | 5′-TGGCCTTCCGTGTTCCTAC-3′ | 5′-GAGTTGCTGTTGAAGTCGCA-3′ |
| Mus *Col1a1* | 5′-AGAGCGGAGAGTACTGGATCG-3′ | 5′-TCGAACGGGAATCCATCGGT-3′ |
| Mus *Fn1* | 5′-AACAGAAATTGACAAGCCGTC-3′ | 5′-TCTGTTTGATCTGGACTGGCA-3′ |
| Mus *Timp1* | 5′-GCAAAGAGCTTTCTCAAAGACC-3′ | 5′-AGGGATAGATAAACAGGGAAACACT-3′ |
| Mus *Zyxin* | 5’-TTTACGCCCCGCAGAAGAAG-3’ | 5’-TGGAGGGGAGGGGAAGATGT-3’ |

**Supporting figures**

**Fig S1.** **β-Hydroxybutyrylation is significantly decreased in bronchiolitis obliterans patient.** Derived from lung histopathological sections of bronchiolitis obliterans patient, Masson’s trichrome staining and IHC staining of acetylation (kac), β-hydroxybutyrylation (kbhb), crotonylation (kcr) and succinylation (ksu). n = 1 (healthy control) or 2 (BO) biologically independent samples.

**Fig S2. Metabolomics analysis demonstrated that the metabolite β-hydroxybutyrylcarnitine was significantly reduced in lung tissues of Bleomycin (BLM) model mice. A** Orthogonal partial least squares discriminant analysis (OPLS-DA) diagram; **B** VIP score plot with the top 15 features ranked by their VIP scores; **C, D** Heatmap of the Glycerophospholipid metabolism and Purine metabolism pathways. In (**A-D**), n = 5 biologically independent samples. *P*-values were obtained using unpaired two-tailed Student’s t-test.

**Fig S3. Transcriptome analysis demonstrating the involvement of** **focal adhesion (FA) pathway and PI3K/AKT pathway in the regulation of pulmonary fibrosis by β-hydroxybutyrylation.** **A** Venn diagrams of the upregulation of BLM/PBS (Fig 4B) and downregulation of BLM-β-OHB/BLM (Fig 4C) genes in the FA pathway and PI3K/AKT pathway highlight the significant intersection; **B** Heatmap of intersecting genes in Supplementary Fig 3A; **C, D** GO analysis of the BLM/PBS and BLM-βOHB/BLM. In (**A-D**), n = 6 (PBS, BLM) or 7 (BLM+β-OHB) biologically independent samples.

**Fig S4. qPCR analysis of Zyxin mRNA expression in mouse lung tissues and mouse lung fibroblast cell line Mlg and macrophage cell line RAW264.7. A** Zyxin mRNA expression in NC-siRNA- and Zyxin-siRNA-transfected mouse lungs as determined by quantitative PCR. **B** qPCR analysis revealed significantly reduced Zyxin mRNA levels in MLg cells following transfection with Zyxin-specific siRNA (si-Zyxin) compared to negative control (si-NC). **C** Zyxin mRNA expression in MLg and RAW264.7 cells as determined by qPCR analysis. In (**A-C**), data are shown as the mean ± SEM, n = 2 (**A**) or 3 (**B, C**) biologically independent samples. *P*-values were obtained using unpaired two-tailed Student’s t-test. **P* < 0.05, ***P* < 0.01, ****P* < 0.001, *****P* < 0.0001.
