# Supplementary material for: The β-hydroxybutyrylation of Zyxin ameliorates pulmonary fibrosis by inhibiting lung fibroblast activation through the PI3K/AKT pathway
Source: Respir Res. 2026 May 30;27:308. doi: 10.1186/s12931-026-03732-0 (PMC13479915; doi:10.1186/s12931-026-03732-0)
Supplement: Supplementary file 2 [file 12931_2026_3732_MOESM2_ESM.docx]

**Original Data Files**

**Fig 1D** Western blot analysis of α-SMA expression in PBS (control) and BLM-treated lung tissues. **Fig 1F** Western blot (Kac: acetylation; Kbhb: β-Hydroxybutyrylation; Kcr: crotonylation; Ksu: succinylation). **Fig 3K** Western blot of fibrosis markers in BLM-induced mice with or without β-OHB.
